# Mapping the interaction site and effect of the Siglec-9 inflammatory biomarker on human primary amine oxidase

**DOI:** 10.1038/s41598-018-20618-4

**Published:** 2018-02-01

**Authors:** Leonor Lopes de Carvalho, Heli Elovaara, Jerôme de Ruyck, Gerard Vergoten, Sirpa Jalkanen, Gabriela Guédez, Tiina A. Salminen

**Affiliations:** 1 Structural Bioinformatics Laboratory, Biochemistry, Faculty of Science and Engineering, Åbo Akademi University, Tykistökatu 6A, FI-20520 Turku, Finland; 2 MediCity Research Laboratory, University of Turku, Tykistökatu 6A, FI-20520 Turku, Finland; 3 University of Lille, CNRS UMR8576 UGSF, F-59000 Lille, France; 4 Centre de Biochimie Structurale, INSERM U554, CNRS UMR 5048, UM1, 29 Rue de Navacelles, 34060 Montpellier Cedex, France

## Abstract

Human primary amine oxidase (hAOC3), also known as vascular adhesion protein 1, mediates leukocyte rolling and trafficking to sites of inflammation by a multistep adhesion cascade. hAOC3 is absent on the endothelium of normal tissues and is kept upregulated during inflammatory conditions, which is an applicable advantage for imaging inflammatory diseases. Sialic acid binding immunoglobulin like-lectin 9 (Siglec-9) is a leukocyte ligand for hAOC3. The peptide (CARLSLSWRGLTLCPSK) based on the region of Siglec-9 that interacts with hAOC3, can be used as a specific tracer for hAOC3-targeted imaging of inflammation using Positron Emission Tomography (PET). In the present study, we show that the Siglec-9 peptide binds to hAOC3 and triggers its amine oxidase activity towards benzylamine. Furthermore, the hAOC3 inhibitors semicarbazide and imidazole reduce the binding of wild type and Arg/Ala mutated Siglec-9 peptides to hAOC3. Molecular docking of the Siglec-9 peptide is in accordance with the experimental results and predicts that the R3 residue in the peptide interacts in the catalytic site of hAOC3 when the topaquinone cofactor is in the non-catalytic on-copper conformation. The predicted binding mode of Siglec-9 peptide to hAOC3 is supported by the PET studies using rodent, rabbit and pig AOC3 proteins.

## Introduction

Inflammatory cascade entails migration of cells such as leukocytes from the circulation to the site of infection through a complex series of events. Human primary amine oxidase (hAOC3), also known as vascular adhesion protein 1 (VAP-1), is an endothelial cell molecule involved in leukocyte trafficking from blood into the tissues during inflammatory responses. Human AOC3 is stored in vesicles in the endothelial cells and upon inflammatory stimuli it is expressed on the endothelial cell surface, where it prevails during inflammation (reviewed in Salmi and Jalkanen 2014^1^). This makes hAOC3 a good target for visualizing inflammation. Interestingly, hAOC3 is a copper containing amine oxidase (primary amine oxidase; E.C.1.4.3.21) with enzymatic and adhesive functions. The adhesive function involves the interaction with leukocytes by the action of sialylated carbohydrates found on its surface^2,3^, while the enzymatic function is responsible for the deamination of primary amines such as, aminoacetone and methylamine, to their corresponding aldehyde products via an oxidative reaction producing hydrogen peroxide and ammonia^4^. In fact, the amine oxidase reaction catalysed by hAOC3 changes the expression of some endothelial selectins involved in the leukocyte extravasation cascade^5^. Besides mediating the interaction between hAOC3 and lymphocytes, the N-glycans at Asn592 (N4), Asn618 (N5) and Asn666 (N6), located on the top of the “cap” of hAOC3 regulate the enzymatic activity of hAOC3^3^. When the asparagine residues in the N4-N6 glycosylation sites were mutated to prevent glycosylation, an increase in the hAOC3 enzymatic activity and a reduction of 25–35% in lymphocyte adhesion was observed, suggesting that in addition to these carbohydrates some other elements may be involved in the hAOC3 mediated adhesion of lymphocytes^3^. It was hypothesized that the removal of the sialylated sugars in hAOC3 would have an effect on its charge and it may affect the structural flexibility, consequently altering the enzymatic activity of hAOC3^3^.

Human AOC3 is a 180-kDa protein that folds into a heart-shaped homodimer^6^. Each monomer has three domains, D2, D3 and D4, of which D4 is the most conserved domain. The active site is buried in the D4 domain with the catalytic residues, including 2,4,5–trihydroxyphenylalanine quinone (TPQ, a post-translationally modified tyrosine cofactor topaquinone), the catalytic aspartate (Asp386), and the three histidines coordinating a copper ion (His520, His522 and His684). The TPQ cofactor can adopt two different conformations, an inactive “on-copper” conformation in which the O5 atom of TPQ is directly coordinated to the copper ion, and an active “off-copper” conformation, where the Cα-Cβ bond of TPQ is rotated by 180 degrees and the O5 atom points towards the substrate channel^7^. In the off-copper conformation, the amine substrate reacts with TPQ forming a Schiff base, which is hydrolysed by the general base Asp386, followed by the release of the aldehyde product. Human AOC3 is reactivated by reduction of molecular oxygen while hydrogen peroxide and ammonia are released.

Sialic acid binding immunoglobulin like-lectin 9 (Siglec-9) is a leukocyte membrane-bound receptor that was found to be a leukocyte ligand of hAOC3^8^. Moreover, Siglec-9 peptides targeted to hAOC3 have been used for PET (Positron Emission Tomography) imaging of inflammation and cancer using different animal models^9–12^ and it was originally proposed that the Siglec-9 peptide binds covalently to the TPQ cofactor of hAOC3^8^. According to the recent data obtained using the full Siglec-9 extracellular domain, Siglec-9 is neither a substrate nor inhibitor for hAOC3 but it enhances the catalytic activity of hAOC3 towards the monoamine substrate benzylamine^13^. The interactions between Siglec-9 and the enzymatic groove of hAOC3 are mediated by two arginines located at the C2_2_ domain of Siglec-9, Arg284 and Arg290 (R3 and R9 in the peptide, respectively)^8,13^. Point mutations of R3 and R9 to alanine reduced binding of the Siglec-9 peptide to hAOC3^8^, whereas the mutations of Arg284 and Arg290 to serines in the recombinant Siglec-9 strengthened the interaction of Siglec-9 to hAOC3^13^.

In the present study, we modelled the hAOC3-attached N-glycans and re-evaluated the binding mode of the Siglec-9 peptide (CARLSLSWRGLTLCPSK) to hAOC3, to better understand the nature of peptide/hAOC3 interaction. We show novel experimental data together with molecular docking simulation that illustrates the binding mode of the Siglec-9 peptide in the active site cavity of hAOC3. These results provide valuable knowledge to aid in the improvement of hAOC3-targeted diagnostic tools and therapeutics.

## Results

### Effect of semicarbazide and imidazole in the binding of Siglec-9 peptide to hAOC3

**Figure 1:**
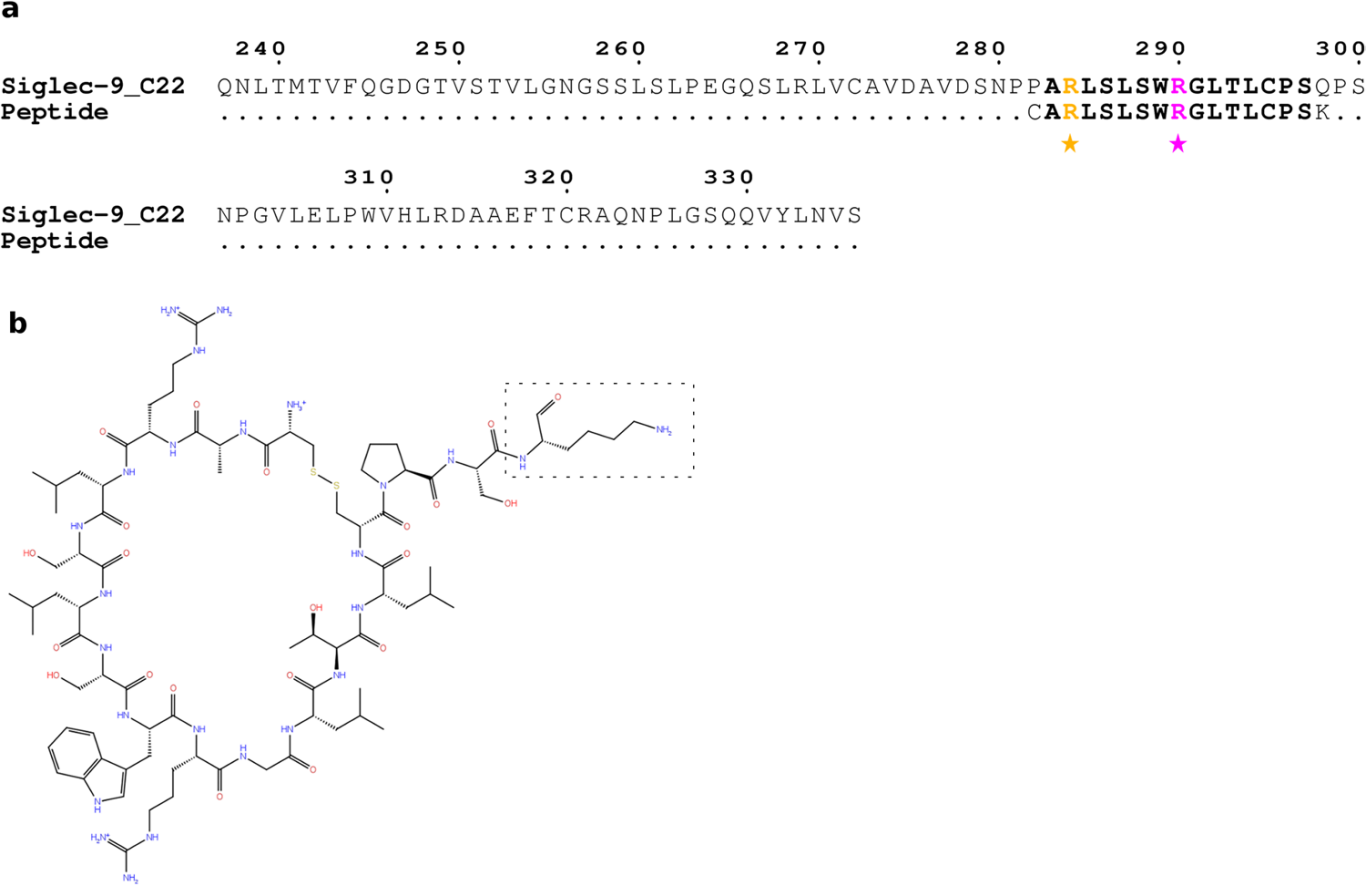
Siglec-9 peptide. (**a**) Sequence alignment of the C2_2_ domain of Siglec-9 and peptide. Coloured in orange is Arg284 (R3) and in magenta Arg290 (R9). The conserved residues between Siglec-9 and the peptide are in bold. The first Cys and terminal Lys in the peptide are the only non-conserved residues. Cys forms a disulfide bridge to Cys14 (Cys295 in Siglec-9) to form the cyclic peptide. Residues in the peptide marked with a star were subjected to mutations. The alignment figure was generated with ESPrit^45^. (**b**) Molecular structure of the Siglec-9 cyclic peptide. The PET tracer is attached to the terminal Lys (dashed box).

**Figure 2:**
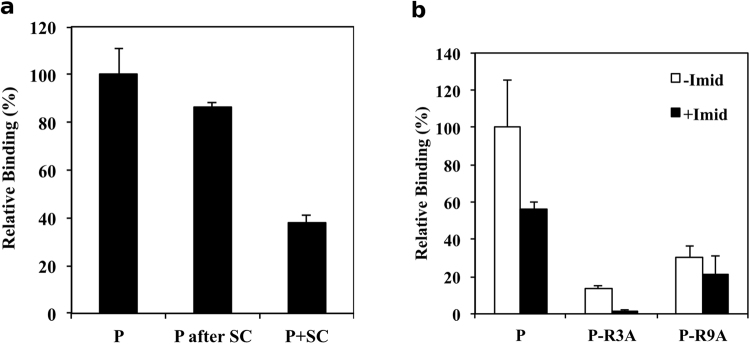
Relative binding of Siglec-9 peptides on immobilized hAOC3 in the presence of semicarbazide and imidazole inhibitors. (**a**) Binding of the Siglec-9 peptide (P, 100%). Injection of 1 mM semicarbazide had no significant effect in peptide/hAOC3 binding (86.1%, P after SC, n = 3, p = 0.188). A mixture of 1 mM SC and Siglec-9 peptide in the same injection reduced the peptide binding to 37.7% (P + SC, n = 3, p = 0.005). (**b**) The presence of 100 mM imidazole reduces the WT peptide binding to 56% when compared to the binding in the absence of imidazole (+Imid, black bars and −Imid, white bars, n = 3, p = 0.0405). Mutation of R3 to Ala decreased the peptide/hAOC3 interaction significantly to 13.6% (−Imid, white bars, n = 2, p = 0.0195) and 1.27% (+Imid, black bars, n = 2, p = 0.0003). Mutation of R9 to Ala decreased binding to 30% (−Imid, white bars, n = 3, p-values = 0.0097) and 21.5% (+Imid, black bars, n = 3, p-values = 0.0039) when compared to the peptide/hAOC3 interaction.

Earlier we have shown that the cyclic Siglec-9 peptide (CARLSLSWRGLTLCPSK) binds to immobilized hAOC3 under flow conditions and that the two arginines R3 and R9 of the peptide are critical for the binding^8^ (Fig. 1). Now we were interested in analysing in more detail how the peptides bind to hAOC3 and study the inhibitory effect of semicarbazide (SC) and imidazole on the peptide/hAOC3 interaction. Semicarbazide is an irreversible inhibitor and binds covalently to the deeply buried TPQ cofactor, whereas imidazole binds reversibly to TPQ but also has a secondary binding site nearer the surface^14^. The results show that blocking the hAOC3 active site with 1 mM SC before injection of the peptide had no significant effect in the peptide binding (Fig. 2a, P after SC, n = 3, p = 0.188). On the other hand, when 1 mM of SC was mixed with Siglec-9 peptide in the same injection, only 37.7% of the peptide bound to hAOC3 (Fig. 2a, P + SC, n = 3, p = 0.005). This suggests that free SC can compete with the peptide binding, and the peptide seems to utilize another binding site in the active site cavity of hAOC3 when SC is covalently attached to the TPQ cofactor.

Since imidazole is known to have an alternative site in the hAOC3 enzymatic groove, we next tested the effect of imidazole in the peptide/hAOC3 interaction by measuring the relative binding of the WT peptide on immobilized hAOC3 in the presence and absence of imidazole. Addition of imidazole reduced the binding to 56% (Fig. 2b, P black bar, n = 3, p = 0.0405). As the peptide/hAOC3 interaction was perturbed by imidazole, we measured the binding of the R3A and R9A mutated Siglec-9 peptides in the presence and absence of imidazole to analyse whether R3 and R9 play a role the interaction of the peptide with hAOC3. The R3A peptide showed only 13.6% binding compared to 100% binding of wild-type peptide in the absence of imidazole, and almost completely abrogated to 1.3% in the presence of imidazole (Fig. 2b, P-R3A compare white and black bars, n = 2, p-values = 0.0195 and 0.0003). Furthermore, the 13.6% reduction in the binding of the R3A peptide was significant (p = 0.0076). These results suggest that R3 mediates the peptide/hAOC3 interaction.

The R9A peptide showed only 30.0% and 21.5% binding to hAOC3 compared to the WT peptide without and with imidazole addition, respectively (Fig. 2b, P-R9A compare white and black bars, n = 2, p-values = 0.0097 and 0.0039). The R3A/R9A peptide showed no binding to hAOC3. Thus, imidazole inhibited the binding of both Arg/Ala mutated peptides to hAOC3.

### Effect of Siglec-9 peptides on the activity of hAOC3

**Figure 3:**
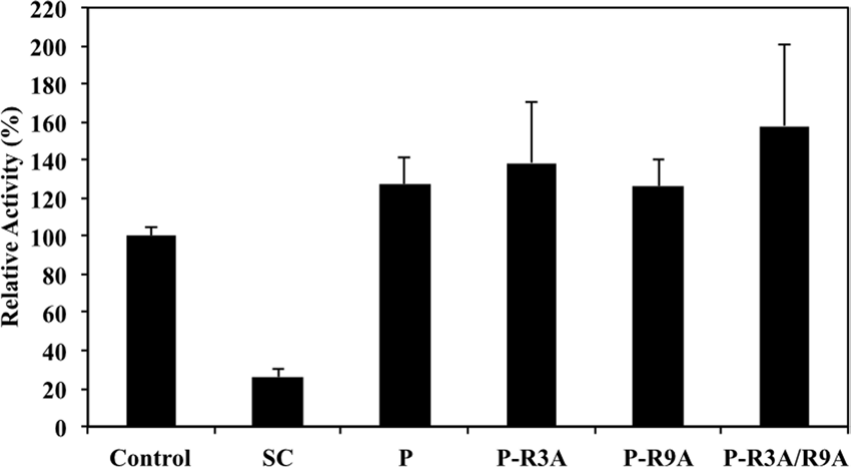
Effect of the Siglec-9 peptides on the enzymatic activity of hAOC3. Human AOC3 activity towards the benzylamine substrate was used as a control for the enzymatic activity (n = 5). The irreversible inhibitor semicarbazide (SC+, n = 5, p = 0.000) reduced the activity whereas the WT Siglec-9 peptide (P, n = 5, p = 0.005) as well as the R3A and R9A mutants (P-R3A and P-R9A, n = 4, p = 0.028 and P = 0.006, respectively) slightly increased the activity. In the presence of the double R3A/R9A mutant (P-R3A/R9A, n = 4, p = 0.019) the enzymatic activity was somewhat higher than in the presence of the other Siglec-9 peptides.

**Figure 4:**
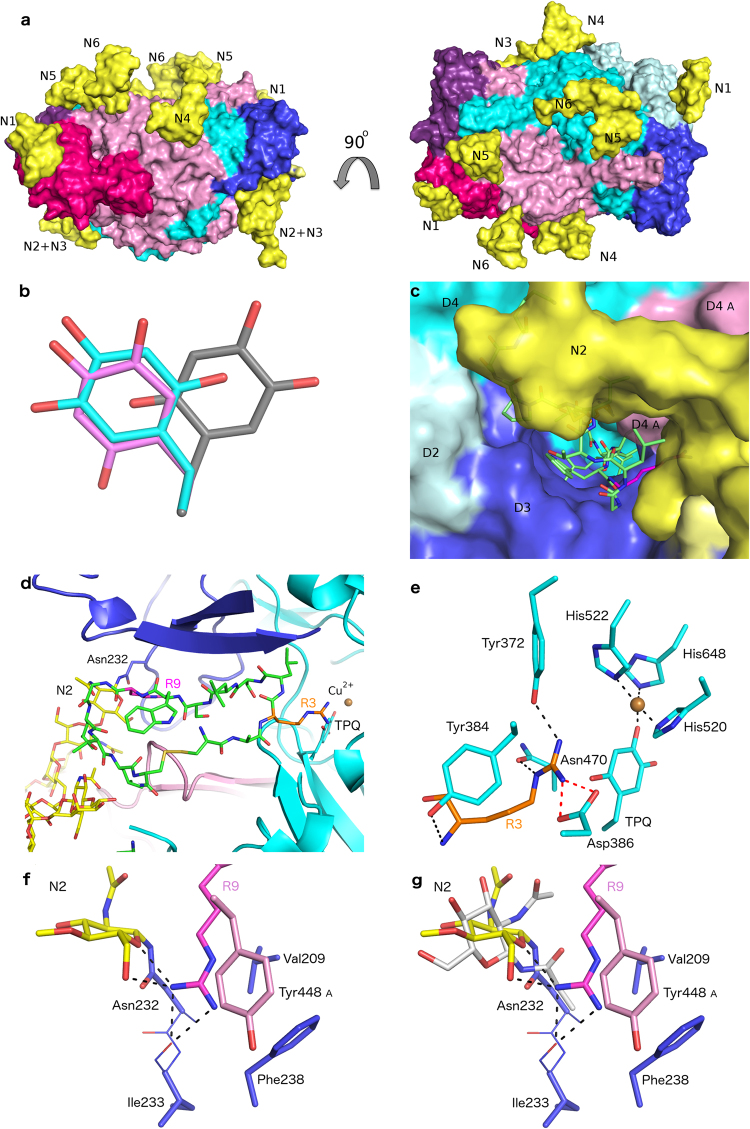
Structural model for the docked complex of N-glycosylated hAOC3 and Siglec-9 peptide. (**a**) Surface representation of hAOC3 with side and top views (90° rotation). Domains D2, D3 and D4 of chain A, are coloured in dark pink, violet, and light pink, respectively, and the same domains in chain B are in pale cyan, blue and cyan. N1 (Asn110) of the modelled glycans (yellow sticks) is located on the edge of the structure, N2 (Asn232) and N3 (Asn294) surround the active site entrance. N4 (Asn592), N5 (Asn618) and N6 (Asn666) are located on top of the structure. (**b**) TPQ conformation. In the crystal structure of hAOC3 (PDB ID 4BTY^16^), TPQ is in the off-copper conformation (gray sticks). After peptide docking, TPQ adopts similar position (pink sticks) as the on-copper TPQ in hAOC3-imidazole complex (PDB ID 2Y73^14^) but the aromatic ring of TPQ is still in the off-copper conformation (cyan sticks). (**c**) Surface view of hAOC3 with docked Siglec-9 peptide. The peptide (green sticks) fills the active site cavity between D3 and D4 of chain B and interacts with the N2 glycan and the tip of β-hairpin arm from D4 of chain A (light pink). (**d**) The docked Siglec-9 peptide occupies the active site cavity of hAOC3 (cartoon). R3 (orange sticks) binds close to TPQ and copper (brown sphere) whereas R9 (magenta sticks) binds near the cavity entrance and the glycosylated Asn232 (N2). (**e**) Close-up view of the interaction mode of R3 (orange). R3 from the peptide forms a salt bridge (red dashes) with Asp386 and hydrogen bonds (black dashes) to Asn470, Tyr372 and Tyr384. (**f**) Close-up view of the interaction mode of R9 (magenta). R9 lies in a pocket formed by N2 and the edge of the active site cavity of hAOC3. It makes hydrogen bonds with the N2 glycan and the backbone of Ile233 as well as hydrophobic interactions with Phe238, Val209 and Tyr448 (chain A). (**g**) Same view as in (**f**) superimposed with the first N-acetylglucosamine unit of the N2 glycan attached to Asn232 in the 4BTY^16^ crystal structure (white sticks).

The extracellular domain of Siglec-9 protein binds to hAOC3 on CHO cells and increases the catalytic activity^13^. Here we tested whether the Siglec-9 peptides have the same effect on hAOC3 activity. We measured the activity of hAOC3 towards benzylamine as positive control (Fig. 3, Control, n = 5). As expected, semicarbazide reduces the enzymatic activity of hAOC3 to about 25% (Fig. 3, SC + , n = 5, p < 0.001). On the contrary, the activity of hAOC3 was slightly increased to 126.8% in the presence of the Siglec-9 peptide, when compared to the control benzylamine-dependent activity (Fig. 4, control vs. P, n = 5, p = 0.005). The R3A and R9A peptides behaved like the WT peptide with 138.6% and 126.3% hAOC3 activity, respectively (Fig. 3, P-R3A and P-R9A, n = 4, p = 0.028 and P = 0.006, respectively), and the R3A/R9A double mutant increased the activity of hAOC3 to 158.0%, slightly more than the R3A and R9A peptides (Fig. 3, P-R3A/R9A, n = 4, p = 0.019). Overall, the hAOC3 activity was not higher than 1.5-fold in the presence of the peptides, suggesting that the Siglec-9 peptides were able to activate hAOC3 but R3 and R9 are not crucial for modulating hAOC3 activity.

### Modeling of N-glycans in hAOC3 and molecular docking of the Siglec-9 peptide

Earlier studies based on Siglec-9 peptides suggested that R3 would mediate the interaction with hAOC3 by interacting with the TPQ cofactor and R9 would have another binding site in the active site cavity^8^. Since the glycans in hAOC3 are important for the enzymatic and adhesive functions of hAOC3^3^, we evaluated the binding mode of the Siglec-9 peptide to hAOC3 and considered for the first time the effect of the N-glycans attached to hAOC3 (Table 1). Based on the model of the fully glycosylated hAOC3, the sugars are distributed throughout the surface of hAOC3 (Fig. 4a). The N1 glycosylation site is located on the edge of the D2 domain, whereas N2 and N3 are located in close proximity to the active site entrance in the D2 and D3 domains, respectively; N4, N5 and N6 are situated on the top of the structure in the D4 domain (Fig. 4a). As the roles of the N2 and N3 glycans are yet unknown, we were specifically interested in finding out how these glycans located near the active site entrance could affect the interaction with the peptide. The Siglec-9 peptide was docked into the active site using GOLD^15^, followed by a post-docking energy minimization to find the best conformation of the hAOC3-Siglec-9 peptide complex and the attached glycans. In the crystal structure of hAOC3 chosen for the docking studies (PDB 4BTY^16^), TPQ is in the catalytically active off-copper conformation, with the C5 atom available for nucleophilic attack of an amine substrate (Fig. 4b). In the docked complex, TPQ has moved to a position where the O4 atom is oriented towards the copper ion, allowing enough space to accommodate the side chain of R3 in the active site (Fig. 4b). As the observed TPQ conformation was compatible with the known intermediate on-copper conformation^17^ (Fig. 4b), we modelled the TPQ to on-copper conformation prior to detailed analysis of the binding mode.

**Table 1:** Summary of the N-glycan structures seen in the crystal structures of AOC3 published to date. HEK293 = Human embryonic kidney cells; residues 29–763; CHO1 = Chinese hamster ovary cells; residues 1–763; CHO2 = Chinese hamster ovary cells; 34–763; NAG = N-acetyl-beta-D-glucosamine; BMA = beta-D-mannose; MAN = alpha-D-mannose; NDG = N-acetyl-alpha-D-glucosamine; FUC = alpha-L-fucose; FUL = beta-L-fucose. ^a^Not linked to the rest of the glycan.

ID	Res./Å	Source	N137	N232	N294	N592	N618	N666	Reference
4BTW:A	2.8	Human serum	NAG-NAG-BMA	NAG-NAG		NAG		NAG	^16^
4BTW:B	2.8	Human serum	NAG-NAG-BMA-MAN-BMA	NAG-NAG		NAG	NAG	NAG	^16^
4BTY:A	3.1	Human serum	NAG-NAG	NAG	NAG	NAG			^16^
4BTY:B	3.1	Human serum	NAG-NAG-BMA-MAN-MAN	NAG-NAG				NAG	^16^
4BTX:A	2.78	Human serum	NAG-NAG-BMA	NAG		NAG			^16^
4BTX:B	2.78	Human serum	NAG-NAG-MAN	NAG	NAG	NAG	NAG	NAG	^16^
2Y73:A	2.6	Human serum	NAG-NAG-MAN	NAG-NAG		NAG		NAG	^14^
2Y73:B	2.6	Human serum	NAG-NAG-MAN	NAG-NAG		NAG		NAG	^14^
2Y74:A	2.95	Human serum	NAG-NAG-MAN	NAG-NAG		NAG		NAG	^14^
2Y74:B	2.95	Human serum	NAG-NAG-MAN	NAG-NAG		NAG		NAG	^14^
2C10:A	2.5	HEK293	NAG-NAG-BMA	NAG	NAG	NGD			^46^
2C10:B	2.5	HEK293	NAG-NAG-BMA	NAG-NAG	NAG	NGD-FUL-NAG-BMA-MAN NAG^a^		NAG	^46^
2C10:C	2.5	HEK293	NAG-NAG-BMA	NAG-NAG	NAG	NGD-FUL-FUL			^46^
2C10:D	2.5	HEK293	NAG-NAG-BMA-MAN	NAG-NAG	NAG	NGD-FUL-NAG-BMA-MAN		NAG	^46^
2C11:A	2.9	HEK293	NAG-NAG	NAG	NAG	NAG-FUL			^46^
2C11:B	2.9	HEK293	NAG-NAG-BMA	NAG	NAG	NGD-FUL-NAG-BMA-MAN		NAG	^46^
2C11:C	2.9	HEK293	NAG-NAG	NAG	NAG	NAG-FUL-NAG			^46^
2C11:D	2.9	HEK293	NAG-NAG-BMA	NAG	NAG	NGD-FUL-NAG-BMA-MAN		NAG	^46^
1US1:A	2.9	CHO	NAG-NAG	NAG					^6^
1US1:B	2.9	CHO	NAG-NAG	NAG					^6^
1PU4:A	3.2	CHO	NAG-NAG	NAG					^6^
1PU4:B	3.2	CHO	NAG-NAG	NAG					^6^
3ALA:A	2.9	CHO	NAG-NAG-BMA	NAG-NAG		NDG-FUL			^47^
3ALA:B	2.9	CHO	NAG-NAG-BMA	NAG		NAG-FUL-NAG			^47^
3ALA:C	2.9	CHO	NAG-NAG-BMA	NAG-NAG		NAG-FUL			^47^
3ALA:D	2.9	CHO	NAG-NAG-BMA	NAG-NAG		NAG-FUL		NAG	^47^
3ALA:E	2.9	CHO	NAG-NAG-BMA	NAG-NAG		NAG-FUL			^47^
3ALA:F	2.9	CHO	NAG-NAG-BMA	NAG-NAG		NAG-FUL			^47^
3ALA:G	2.9	CHO	NAG-NAG-BMA	NAG-NAG		NAG-FUC			^47^

In overall, the docking results presented here show that the Siglec-9 peptide effectively occupies the active site cavity of hAOC3, forming extensive intermolecular interactions with residues from the D3 and D4 domains (Fig. 4c, see also Table 2). Moreover, the peptide interacts with the N2 glycan and with the tip of the long β-hairpin arm of D4 domain from chain A (Fig. 4c). R3 in the peptide protrudes into the active site near TPQ, while R9 interacts near the surface next to the N2 glycan (Fig. 4d). However, R3 does not interact directly with TPQ, instead its guanidinium group forms a salt bridge with the catalytic aspartate (Asp386), hydrogen bonds with the hydroxyl group of Tyr372 and the carbonyl group of Asn470 as well as hydrophobic contacts with Tyr384 (Fig. 4e). The R9 binding site is located in a pocket formed on the surface of hAOC3 and the N2 glycan (Fig. 4f). The guanidinium group of R9 forms hydrogen bonds with the first N-acetylglucosamine unit of the N2 glycan, which is located in a similar position as the corresponding sugar in the X-ray structure (Fig. 4g). Additionally, R9 makes interactions with Val209 and Phe238 as well as Tyr448 from the other monomer (Fig. 4f). The other residues in the peptide are stabilized by main chain hydrogen bonds and hydrophobic interactions with the side chains of Tyr176, Leu177, Asp180, Thr212, Thr213, Phe227 and Tyr372 (Table 2).

**Table 2:** Putative interactions between hAOC3 and the Siglec-9 like peptide (sequence: CARLSLSWRGLTLCPSK) according to the docking. The residues in the active site of different organism used for the PET studies are also listed in the table, with conserved residues in bold. Residues in grey background are not present in the Siglec-9 C2_2_ domain. SC = Side chain; MC = Main chain; H-bond = Hydrogen bond. ^a^Arm I of chain A.

Siglec-9 Peptide (Protein)	hAOC3 Chain B	ratAOC3	mouseAOC3	rabbitAOC3	pigAOC3	Predicted type of peptide- hAOC3 interaction
Residues						
C1	Tyr394	**Tyr**	**Tyr**	**Tyr**	**Tyr**	MC-SC H-bond
A2 (283)	Tyr176	**Tyr**	**Tyr**	**Tyr**	**Tyr**	Hydrophobic
R3 (284)	Tyr384 Asp386 Asn470 Tyr372	**Tyr** **Asp** **Asn** **Tyr**	**Tyr** **Asp** **Asn** **Tyr**	**Tyr** **Asp** **Asn** **Tyr**	**Tyr** **Asp** **Asn** **Tyr**	MC-SC H-bond Hydrophobic Salt bridge SC-SC H-bond SC-SC H-bond
L4 (285)	Tyr372 Thr212 Thr213	**Tyr** **Thr** **Thr**	**Tyr** **Thr** **Thr**	**Tyr** **Thr** Ala	**Tyr** **Thr** Ser	Hydrophobic MC-MC H-bond Hydrophobic
S5 (286)	Asn470	**Asn**	**Asn**	**Asn**	**Asn**	SC-MC H-bond
L6 (287)	Thr212 Phe227	**Thr** **Phe**	**Thr** **Phe**	**Thr** **Phe**	**Thr** **Phe**	Hydrophobic Hydrophobic
W8 (289)	Thr210 Leu177 Asp180	Lys Gln Gln	**Thr** Gln Glu	Val **Leu** **Asp**	**Thr** **Leu** **Asp**	MC-MC H-bond Hydrophobic Hydrophobic
R9 (290)	Val209 Ile233 Phe238 Tyr448^a^	Leu Leu **Phe** **Tyr**	Leu Leu **Phe** **Tyr**	Ala **Ile** **Phe** His	**Val** **Ile** **Phe** Arg	Hydrophobic SC-MC H-bond π-stacking π-stacking
P15 (296)	His762	Tyr	Tyr	**His**	**His**	MC-SC H-bond
S16 (297)	Ser419	**Ser**	**Ser**	**Ser**	**Ser**	MC-SC H-bond

### Comparison of the binding mode of the Siglec-9 peptide and hAOC3 inhibitors

**Figure 5:**
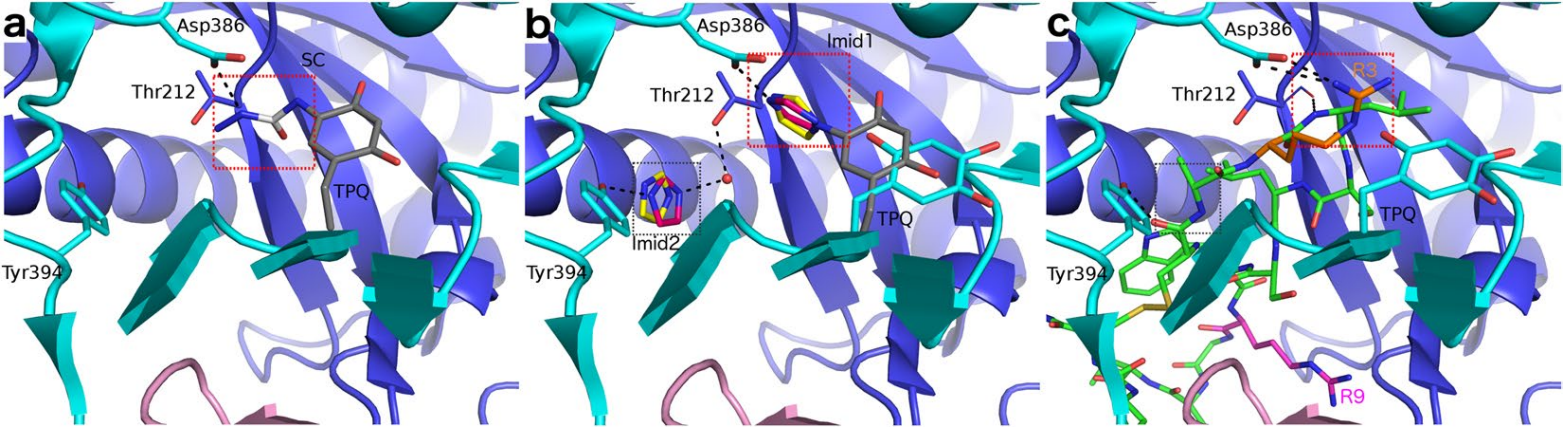
Comparison of the binding modes of semicarbazide, imidazole and the Siglec-9 peptide. (**a**) Semicarbazide (SC, white sticks) covalently docked to TPQ is predicted to from a hydrogen bond (black dashes) to Asp386. (**b**) Human AOC3 has two imidazole binding sites (black and red dashed box) in the active site cavity. The Imid1 (red dashed box) site is buried in the active site of the D4 domain (cyan) and Imid2 (black dashed box) is located in the middle of the active site cavity between the D3 (violet blue) and D4 domains. Imidazole (red sticks) in the off-copper TPQ complex of hAOC3 (white sticks; PDB ID 2Y74) binds in the same site as the imidazole (yellow sticks) in the on-copper complex TPQ (gray sticks; PDB ID 2Y73). (**c**) The Siglec-9 peptide (green sticks) docked into the active site of hAOC3 occupies the same region as the imidazole bindings sites. We suggest that in the alternative-binding mode, R3 (orange sticks) from the peptide could bind near the Imid2 (black dashed box) binding site. The copper ion is represented as an orange sphere and R9 as magenta sticks, hydrogen bonds and salt bridge as black dashes.

The binding analysis presented in this work suggests that semicarbazide and imidazole can compete with the Siglec-9 peptide for the same binding site in hAOC3 (Fig. 2). To compare the binding mode between the peptide and the irreversibly binding semicarbazide, the inhibitor was covalently docked into the C5 atom of the off-copper TPQ in the crystallographic structure of hAOC3. The results show that semicarbazide forms a hydrogen bond with the catalytic base Asp386 (Fig. 5a). This is in agreement with the activity assays, in which hAOC3 activity was practically lost in the presence of semicarbazide (Fig. 3). The crystal structures of hAOC3-imidazole complexes showed that imidazole binds to both the on-copper (PDB ID 2Y73) and off-copper (PDB ID 2Y74) conformations and has two binding sites in the active site cavity of hAOC3 (Fig. 5b)^14^. Comparison of the binding mode between docked semicarbazide, imidazole in the complex structures and the Siglec-9 peptide reveals that R3 similarly interacts with Asp386 and occupies the same region in the active site of hAOC3 as these inhibitors (Fig. 5a–c, position of SC or Imid1, red box). Furthermore, Tyr394 and Thr212 involved in imidazole binding (Fig. 5b) interact with the main chain atom of the docked Siglec-9 peptide (Fig. 5c; Table 2). Interestingly, the binding of the Siglec-9 peptide was not significantly affected when semicarbazide was added prior to the peptide (Fig. 2, P after SC), suggesting that R3 binds to a different site, presumably the second imidazole-binding site (Fig. 5b, position of Imid2, black box) when its access to TPQ is blocked by semicarbazide.

### Comparison of the docking results with the PET preclinical studies using Siglec-9 peptide

To date, the Siglec-9 peptide (CARLSLSWRGLTLCPSK) has been used as a AOC3-targeted tracer in animal models, such as rats^8,11,18–21^, rabbits^9^, pigs^12^, transgenic hAOC3 and non transgenic mice^8^, and recently in blood samples of human, rat, rabbit and pig^10^. The Siglec-9 peptide based PET imaging in the experimental models with tumour-bearing and inflammatory manifestations show positive signal in injured tissues. To study the conservation of the residues predicted to be involved in the binding of the Siglec-9 peptide, we compared the sequences of AOC3s from human and the species used in PET studies (Supplementary Fig. S1).

The overall sequence identity of hAOC3 is 82% to mouse, 83% to rat, 84% to rabbit, and 85% to porcine AOC3 (Fig. S1). Comparison of the residues predicted to be involved in the Siglec-9 peptide/hAOC3 interaction with the corresponding residues from mouse, rat, rabbit and pig AOC3s shows that these residues in general are highly conserved or conservatively replaced (Fig. S1). The residues in contact with R3 are totally conserved (Fig. 4e, Table 2) suggesting that similar interactions occur in mouse, rat, rabbit and pig AOC3s. Of the residues interacting with R9, Phe238 is conserved and Val209 is either conserved (porcine AOC3) or replaced by a hydrophobic Ala (rabbit) or Leu (rodents) (Fig. 4f, Table 2). Tyr448 is replaced by a His and by an Arg in rabbit and pig AOC3, respectively (Fig. 5f, Table 2), which may form His-Arg and Arg-Arg stacking^22–26^ interactions with the guanidinium of R9. Furthermore, the hydrogen bond between R9 and the backbone of Ile233 (replaced by a Leu in rodent AOC3s) could similarly occur in all species. The Asn corresponding to Asn232 (N2) in hAOC3 (Fig. 4f) is likely glycosylated in the other species and, thus, the attached glycan may interact with R9 of the Siglec-9 peptide (Fig. 4f). While the rabbit AOC3 lacks the corresponding N-glycosylation motif (see alignment Fig. S1), the interaction of the Siglec-9 peptide likely occurs solely with the protein. Additionally, Leu177 and Asp180 interacting with Trp8 in the peptide are replaced by two Gln (in rat AOC3) or Glu and Gln (in mouse AOC3) (Table 2). As the hydrophobic parts of these residues form hydrophobic interactions with Trp8, the differences are not likely to have a major effect on the peptide/AOC3 interaction. Taking together, the residues involved in Siglec-9 binding, in particular those in contact with R3, show a high degree of conservation in AOC3 from human, rat, mouse, rabbit and pig.

## Discussion

Human AOC3 plays a role in the recruitment of leukocytes to sites of inflammation. Accumulating data support the suitability of hAOC3 as a target for *in vivo* imaging of inflammation using Siglec-9 peptides as tracers^8–11,27^. We report here the docked Siglec-9 peptide/hAOC3 complex that is consistent with the biochemical and interaction studies presented in this work and in the previous studies using the extra cellular domain of Siglec-9^13^. In accordance with Aalto *et al*.^8^, our docking studies show that both R3 and R9 in the peptide interact with the glycosylated hAOC3. R3 makes contacts with the catalytic region of hAOC3, and R9 binds in a pocket formed by the N2 glycan and the hAOC3 surface (Fig. 4d).

In accordance with our previous results with the Siglec-9 protein^13^, the results presented here show that the Siglec-9 peptide, in particular the R3 residue, competes for the same binding site as the semicarbazide and imidazole inhibitors (Figs 2 and 5). Despite using a crystal structure of hAOC3 with the off-copper catalytic form of TPQ^28^, the docking simulation resulted in a TPQ conformation similar to the non-catalytic on-copper form, but the orientation of the TPQ aromatic ring was similar to the intermediate on-copper conformation^17^. Our results propose that the binding of R3 to the catalytic site of hAOC3 may take place when TPQ is in the on-copper conformation, as it would not fit well into the same site when TPQ is in off-copper conformation (Fig. 5c). Furthermore, the predicted binding mode to the on-copper conformation is in agreement with the fact that the Siglec-9 peptide is not a hAOC3 substrate^8^, which bind to the productive off-copper TPQ conformation. The mechanism that regulates the conformational changes of TPQ is not yet understood. It has been shown that the cofactor can change its conformation during the reductive half-reaction in the presence of various amines substrates^17^. Furthermore, in the crystal structures of hAOC3-imidazole complexes TPQ alternatively adopts the on-copper (PDB ID 2Y73) or the off-copper (PDB ID 2Y74) conformation (Fig. 5b)^14^. In a similar way as the predicted binding mode of Siglec-9 peptide, berenil and pentamidine inhibitors of human diamine oxidase (hDAO or hAOC1; diamine oxidase; E.C.1.4.3.22) interact with hDAO when TPQ is in the on-copper conformation^29^.

Neither the peptide^8^ nor the Siglec-9 protein^13^ are substrates for hAOC3 but both of them similarly enhance the enzymatic activity of hAOC3. As the active site of copper containing amine oxidases is buried in the D4 domain, conformational changes of the D2 and D3 domains lying on the surface of D4 have been suggested to enhance substrate access to the active site^30^. Since injection of semicarbazide before the Siglec-9 peptide did not inhibit the peptide binding, we suggest that R3 might bind to the same site as the secondary imidazole (Imid2) in the middle of the active site channel of hAOC3 when the TPQ-bound semicarbazide blocks R3 access to the active site of hAOC3, and the docking results support this hypothesis (Fig. 5a and c). The Siglec-9 peptide would enhance the catalytic reaction of hAOC3 by also binding to hAOC3 when the TPQ is in the off-copper conformation without blocking substrate access to TPQ. Therefore, binding of Siglec-9 peptide (this study) and the Siglec-9 protein^13^ to hAOC3 might induce conformational changes on the D2 and D3 domains, to allow easier access for substrate to the catalytic site of hAOC3 enhancing the amine oxidase activity of hAOC3.

Preclinical studies are made using animal models, as it allows performing the studies in a way that is not possible using humans. Comparison of the hAOC3 residues involved in hAOC3/Siglec-9 peptide interaction with AOC3 proteins from pig and rabbit indicates that the differences in the active site channel of AOC3s might affect the interaction mode of the Siglec-9 peptide and, thus, the PET studies in these species. Although the R3 binding site in the docked hAOC3/peptide complex is totally conserved in the studied species, the differences especially in the R9 binding site of pig and rabbit AOC3 might weaken the Siglec-9 peptide binding. Furthermore, rabbit hAOC3 lacks the N2 glycosylation site and, thus, it cannot make glycan - R9 interactions. The peptide binding has been reported to be lower to pig and rabbit AOC3 than to human or rodent proteins^10^, which is consistent with our comparison of the differences in the interacting residues and further supports our model for peptide/AOC3 interactions. Unlike rat, mouse and human, rabbit and pig have high serum amine oxidase activity as a result of the additional AOC protein encoded by the *AOC4* gene^31^. In fact, a recent study using plasma from different mammals shows species-specific differences in the binding of the Siglec-9 peptide^10^. Altogether, we suggest that the usage of organisms with high serum level might cause problems, as the active site of AOC3 in these species seems to differ from hAOC3 more than that of rodent AOC3s. Furthermore, Jensen *et al*. (2017)^10^ reported that the *in vivo* binding of the PET peptide to pig plasma was higher than *in vitro* binding, which suggests that the AOC4 protein might influence the *in vivo* results. Moreover, there is accumulating evidence that species-specific differences affect ligand recognition and binding. For instance, inhibitors developed for hAOC3 have failed to bind or have weaker potency towards rodent AOC3^16,32^. Also different amine substrates have different kinetic behaviour in human and mouse AOC3s^33^. Structural comparison between hAOC3 and AOC3 proteins from mouse, rat and monkey reveals that despite the high sequence identity, differences in the active site channel most likely result in different species-specific ligand recognition. Thus, the species-specific differences should always be taken into account when designing large inhibitors and diagnostic peptides.

Peptides generally provide a good model for binding studies of protein-protein interactions, as they can mimic the features of the protein of interest and are easier to synthesize^34,35^. In conclusion, the results obtained here with the Siglec-9 peptides are consistent with our previous studies using the full-length extracellular region of Siglec-9^13^ suggesting that the binding mode of both, protein and peptides, are most likely similar. Further experimental analysis of the Siglec-9 peptide interaction with hAOC3 will likely enlighten the binding characteristics of Siglec-9 protein.

## Materials and Methods

### Materials

All the reagents were from Sigma-Aldrich unless otherwise mentioned. The cyclic Siglec-9 peptide (CARLSLSWRGLTLCPSK) was designed to match the predicted loop in Siglec-9 C2_2_ domain corresponding to residues 283 to 297 (ARLSLSWRGLTLC) (Fig. 1a and b). Similar Siglec-9 peptides with mutations in R3, R9 and R3/R9 into alanine (CAALSLSWRGLTLCPSK, CARLSLSWAGLTLCPSK and CAALSLSWAGLTLCPSK, respectively; hereafter called R3A, R9A and R3A/R9A peptides) were also used (corresponding to Arg284, Arg290 and Arg284/Arg290 in Siglec-9: Fig. 1a). The peptides were purchased from Peptides International (Kentucky, USA) or from PopyPeptide Group (Strasbourg, France) dissolved in water and further diluted to an appropriate buffer for the experiments. Human recombinant AOC3 was produced in Chinese hamster ovary (CHO) cells and purified by immunoaffinity chromatography as described earlier^2,13,36^.

### Binding studies by surface plasmon resonance

The binding of cyclic Siglec-9 peptides to immobilized recombinant hAOC3 was studied by surface plasmon resonance (SRP) similarly as earlier described^8^. Human AOC3 was coupled to a CM5 sensor chip (GE) via amine coupling according to manufacturer’s instructions. For the coupling step, hAOC3 was diluted with 10 mM Na-acetate buffer pH 4.5 to a concentration of 50–200 nM. The coupling resulted to about 5000–8000 RU of bound hAOC3 (5000–8000 pg/mm^2^). The high level of coupled-hAOC3 was chosen based on the binding assays of extracellular Siglec-9 protein on immobilized hAOC3 described before^13^. The binding of 100 µM Siglec-9 peptide to hAOC3 was studied using running buffer containing 10 mM HEPES, 150 mM NaCl, pH 7.4 with 0.005% P20 surfactant. The peptide was diluted in the same buffer. We studied the binding of peptide for 1 min using 20 µl/min flow and monitored the binding at the end of the injection. The effect of imidazole on binding was studied by addition of 100 mM imidazole in the peptide solution. The effect of irreversible semicarbazide inhibitor on the binding of Siglec-9 peptide was monitored before and after 5 min injection of 1 mM semicarbazide over the immobilized hAOC3 or including 1 mM SC in the peptide sample. The concentrations of inhibitors were chosen according to previous studies, where we have shown that 100 mM imidazole and 1 mM semicarbazide concentration inhibits the activity of hAOC3^13^. The experiments were repeated 2–3 times.

### Activity assay

The effect of Siglec-9 peptides on the activity of hAOC3-expressing CHO cells^37^ was studied as described in^27,37^. In brief, the water-solubilized peptide was added on the cells covered in reaction buffer (20 mM HEPES, 5 mM KH_2_PO_4_, 1 mM MgSO_4_, 1 mM CaCl_2_, 136 mM NaCl, and 4.7 mM KCl, pH 7.4) to a final concentration of 5 μM and incubated at 37 °C/5% CO_2_ for 30 min. The radioactive substrate [7–14 C]benzylamine (Amersham Pharmacia, 54 mCi/mmol) was added at 2.5 μM and the cells were incubated at 37 °C/5% CO_2_ further 2 hrs. The reaction was stopped with 2 M citric acid and the formed, labeled benzaldehyde was extracted to *p*-toluene, and the activity of organic phase was determined (Wallac-1409 Liquid scintillator, Wallac, Turku, Finland). For every condition we used triplicates, and the experiments were repeated 4–5 times.

### Statistics

We used the Student *t* test for the comparison of means. The p-values below 0.05 were considered significant.

### Modeling of glycans in hAOC3

**Figure 6:**
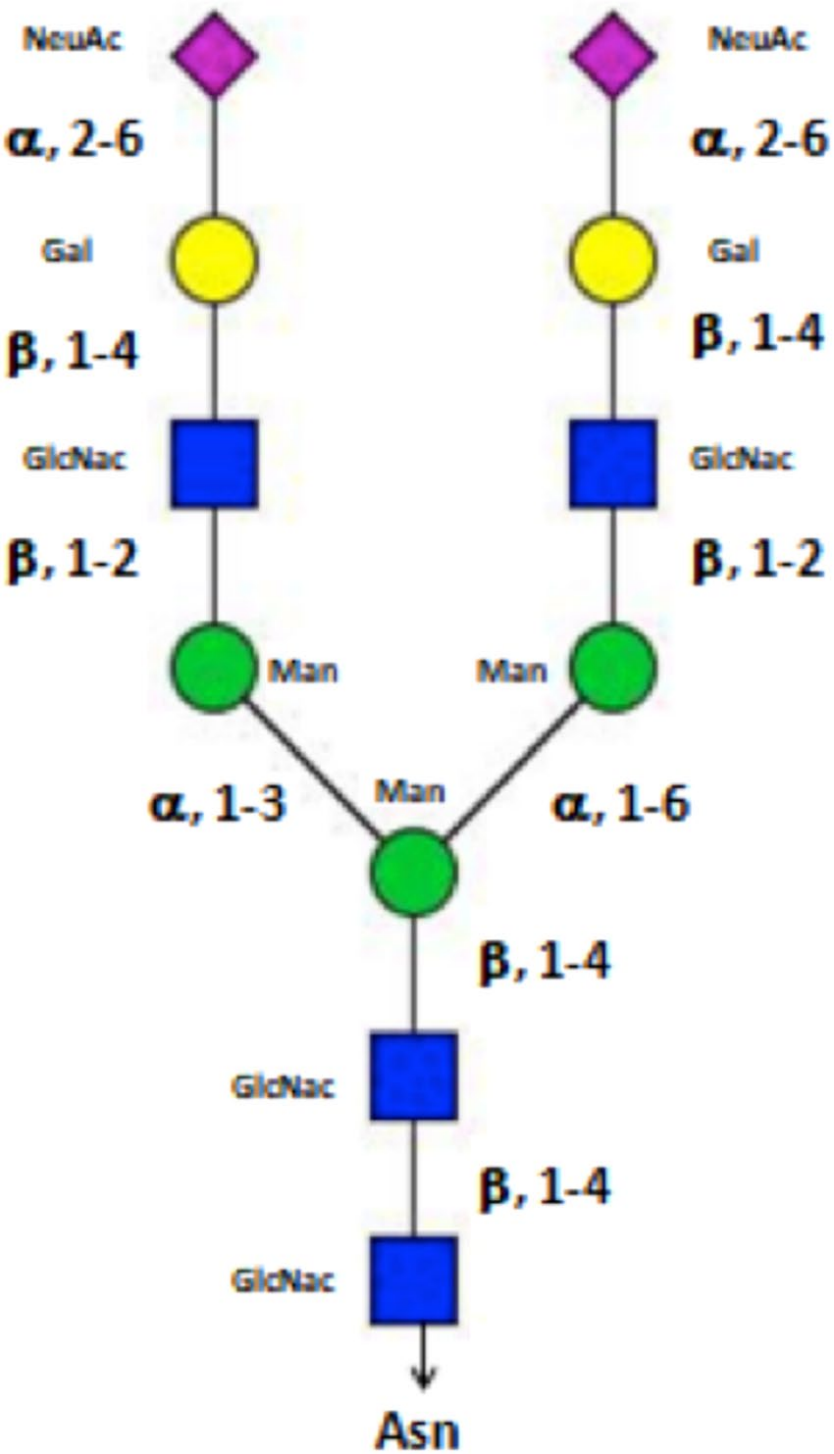
Schematic presentation of the modelled glycans in hAOC3. The glycoforms are illustrated for N-acetylglucosamine, mannose, galactose and N-acetylneuraminic acid. A fucose can be branched on the first N-acetylglucosamine, as observed at position N592 (N4) in several hAOC3 crystal structures (see Table 1).

Sialic acids are known to be crucial for the hAOC3-mediated adhesion^2,3^. As all six N-glycosylation sites are known to have attached glycans^3^ we modelled complex type N-glycans (Fig. 6) to the six Asn residues of both chains, all of which have at least one attached sugar unit in the known X-ray structures of hAOC3 (Table 1). The crystal structure of hAOC3 in complex with the largest pyridazinone (PDB ID 4BTY^16^) was chosen for the study as it is the natural form of hAOC3 extracted from human serum and all the C-terminal residues located near the active site entrance are visible only in this structure. Despite there are no major conformational changes in hAOC3 upon ligand binding, the rotamer of Phe173 in the chosen structure makes the cavity slightly more accessible compared to the other than pyridazinone complex structures (PDB ID 4BTW and PDB ID 4BTX^16^). Furthermore, the selected structure has some sugar units attached to N1 (Asn137), N2 (Asn232), N3 (Asn294), N4 (Asn592) and N6 (Asn666) in at least one of the chains (Table 1). Despite, no sugar is observed at N5 (Asn618), the N5 site is glycosylated in some of the structures (Table 1). The true nature of the hAOC3 sugars is unknown; however, the common core sugar sequence shared by all N-glycans, Manα1–6(Manα1–3)Manβ1–4GlcNAcβ1–4GlcNAcβ1-Asn is resolved for Asn137 in chain B of the X-ray structure (Table 1; PDB ID 4BTY^16^). Thus, the complex type N-glycans were built in the hAOC3 X-ray structure using BIOVIA Discover Studio v4.5 (San Diego, CA, www.accelrys.com). Additionally, a fucose residue was added to the first GlcNAc at N4 (Asn592) site, as it was present in several crystal structures of hAOC3 (Table 1). A Monte-Carlo sampling was then applied to the subsequent structure, in order to cover the conformational spaces of the complete glycans. The use of this technique, instead of molecular dynamics, was favored because of its ease of use and its rapidity as is also well describe by Jorgensen and Tirado-Rives^38^. The identification of the putative binding mode of the ligand inside the active site of the protein was performed on a set of glycan conformation reflecting the dynamic part of this segment. The lowest energy structure was kept as the target protein for the docking procedure.

### Computational analysis and peptide docking

The cyclic Siglec-9 peptide (Fig. 1) used as ligand of hAOC3 in the docking was generated using BIOVIA Discover Studio v4.5 (San Diego, CA, www.accelrys.com). The docking experiments were performed with GOLD (Genetic Optimization for Ligand Docking)^15^ version 5.2. The active site of hAOC3 (reference structure PDB ID 4BTY^16^) with the modelled N-glycans was defined covering a 15 Å radius sphere centred at the O5 atom of TPQ in chain B, a distance that also extended to chain A. The Siglec-9 peptide was considered as fully flexible during the docking process. Residues that were included in this sphere were considered flexible and a genetic algorithm with default parameters was applied during the whole process^15^. The side chains of residues in the vicinity of the active site were thus defined as flexible during the docking procedure (chain A: Arg442, Asp446, Leu447, and Tyr448; chain B: Phe239, Tyr384, Asp472, Trp475, Asp476 and Phe489). Poses were selected based on the computed ligand receptor interaction energy of each pose. The best 20 poses ranked according to the ChemPLP fitness scoring function in GOLD were kept for further analysis. The 20 poses were analysed taking into account previously published work on the Siglec-9 peptide^8^, the extracellular part of Siglec-9^13^ and the hAOC3 structures (PDB entries 2Y73, 2Y74 and 4BTY^14,16^), which limits the possible poses to those where both R3 and R9 interact with the active site cavity, R3 binds near the TPQ cofactor and the terminal Lys is located near the protein surface. As a result, the highest ranked pose fitted best with previous knowledge and was selected as a representative pose for the peptide/hAOC3 complex. The final peptide/hAOC3 complex was energetically minimized by molecular mechanics using the Spectroscopic Empirical Potential Energy function SPASIBA force field with the default parameters (see^39,40^). This force field is well adapted to flexible structure, as it is parameterized with dynamic data such as molecular vibrations. Interestingly, during the minimization process, the TPQ rotates from the off-copper conformation to an intermediate on-copper conformation, which Murakawa *et al*. recently revealed upon the movement of TPQ from the off-copper to on-copper conformation^17^. In the intermediate on-copper conformation, the TPQ ring has slid by ~53° rotation around the Cα–Cβ bond and tilted up by ~ 20° rigid body rotation centered at the Cα carbon prior to the final revolution by 180° rotation around the Cβ–Cγ bond to the on-copper conformation^17^. This suggests that the peptide in the docked mode binds to on-copper TPQ and, thus, TPQ was adjusted to the on-copper conformation in the final peptide/hAOC3 complex. The predicted type of interactions between Siglec-9 peptide with hAOC3 were determined by the PLIP server^41^ and using Maestro (Schrödinger, LLC), which list non-covalent protein–ligand interactions such as hydrophobic interactions, hydrogen bonds, salt bridges and π-stacking. Illustrative pictures of the peptide/hAOC3 complex were generated using PyMol^42^.

### Sequence analysis

Protein sequences from hAOC3, rat (*Rattus norvegicus*) (ID: O08590), mouse (*Mus musculus*) (ID: O70423) AOC3s were collected from UniProtKB^43^ and sequences pig (*Sus scrofa*) (ID: XP_020922632.1) and rabbit (*Oryctolagus cuniculus*) (ID: XP_002719460.1) from NCBI (www.ncbi.nlm.nih.gov). These sequences were aligned using the MALIGN tool in BODIL^44^.

### Data availability

The authors declare that all data supporting the findings of this study are available in the article (and in Supplementary Information).

## Electronic supplementary material

Supplementary Information
