# Supplementary material for: Mapping the interaction site and effect of the Siglec-9 inflammatory biomarker on human primary amine oxidase
Source: Sci Rep. 2018 Feb 1;8:2086. doi: 10.1038/s41598-018-20618-4 (PMC5794975; doi:10.1038/s41598-018-20618-4)
Supplement: Supplementary file 1 — Supplementary Information [file 41598_2018_20618_MOESM1_ESM.pdf]

Mapping the interaction site and effect of the Siglec-9 inflammatory biomarker on human primary amine oxidase

Supporting Information

Leonor Lopes de Carvalho[1], Heli Elovaara [2], Jerôme de Ruyck[3,4], Gerard Vergoten[3], Sirpa Jalkanen[2], Gabriela Guédez[1], Tiina A. Salminen[1*]

[1] Structural Bioinformatics Laboratory, Biochemistry, Faculty of Science and Engineering, Åbo Akademi University, Tykistökatu 6A, FI-20520 Turku, Finland

[2] MediCity Research Laboratory, University of Turku, Tykistökatu 6A, FI-20520 Turku, Finland

[3] University of Lille, CNRS UMR8576 UGSF, F-59000 Lille, France.

[4] Centre de Biochimie Structurale, INSERM U554, CNRS UMR 5048, UM1, 29 Rue de Navacelles, 34060 Montpellier Cedex, France.

* Corresponding author: tiina.salminen@abo.fi

Supplementary Figure S1: Sequence alignment of AOC3 proteins from human, pig, rabbit and rat. The active site motif is highlighted with a magenta box and the Asn232 N-glycosylation motif with a blue box. Red stars show the residues predicted to be involved in the peptide binding. Image from the sequence alignment was generated in ESPrit[1].

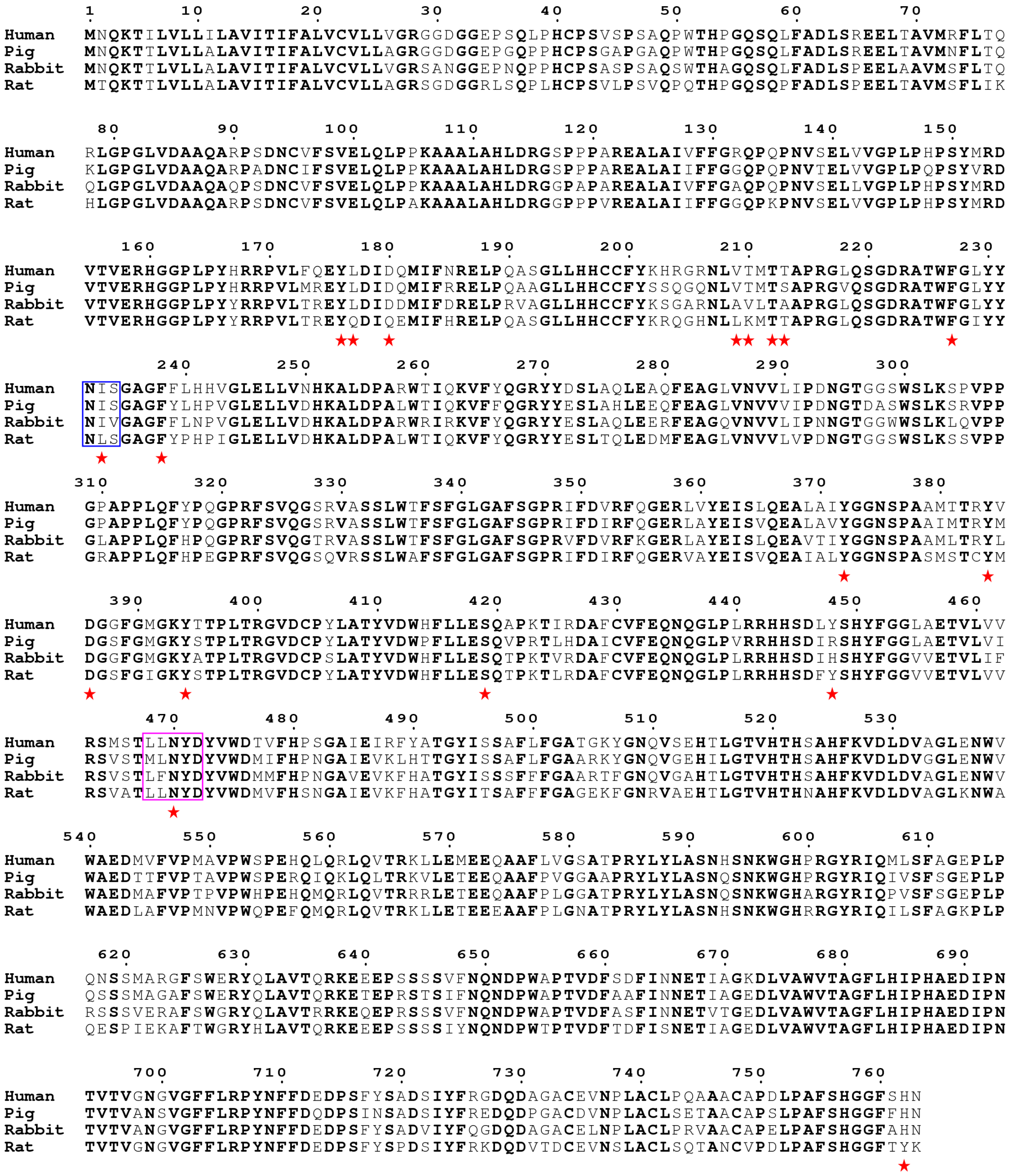

References

1. Robert, X. & Gouet, P. Deciphering key features in protein structures with the new ENDscript server. *Nucleic Acids Res.* **42,** 320–324 (2014).
